# Innervation of nociceptors in intact human menisci along the longitudinal axis: semi-quantitative histological evaluation and clinical implications

**DOI:** 10.1186/s12891-019-2706-x

**Published:** 2019-07-22

**Authors:** Yipeng Lin, Kaibo Zhang, Qi Li, Jian Li, Bin Xu

**Affiliations:** 1 Department of Orthopaedic Surgery, West China Hospital, Sichuan University, Chengdu, People’s Republic of China 610041; 2 West China School of Public Health, Sichuan University, Chengdu, China

**Keywords:** Meniscus, Nociceptor, Immunohistochemistry, Quantitative assessment, Gold chloride staining

## Abstract

**Background:**

The mechanism of pain after meniscus injury remains unknown. After injury, some individuals suffered from acute pain, while others suffer from delayed pain. A precise nociceptor distribution pattern may provide the answer to this question.

**Methods:**

Twenty-two intact menisci (paired medial and lateral menisci) were obtained from 11 patients with a mean age of 28.45 years. All menisci were sectioned into five parts: the anterior horn, anterior body, middle body, posterior body, and posterior horn. Two paired menisci were stained by a modified gold chloride method. All other specimens were stained by H&E staining and were subjected to immunohistochemical staining to detect substance-P (SP). Under a microscope, measurements were made in 10 consecutive visual areas at 400x magnification. SP-positive fibres were determined using a three-grade scale, and the mean number of SP-positive fibres was assessed.

**Results:**

Nerve fibres and nociceptors stained by H&E and modified gold chloride were found mainly in the vascular outer third of the menisci as observed under a microscope; the positive area was wider in the anterior and posterior horns. There were more SP+ fibres in the anterior horn and posterior horn than in the anterior body, middle body, or posterior body (*p* < 0.05). Regarding the bodies, the mean number of substance-P fibres was greater in the anterior body or posterior body than in the middle area (*p* < 0.05). No significant differences were found between the number of substance-P nerve fibres in the anterior horn vs the posterior horn or in the anterior body vs the posterior body of all menisci (*p* > 0.05). No significant differences were observed in the same location between the paired medial and lateral menisci in all areas of the menisci (*p* > 0.05).

**Conclusion:**

The density of nociceptors decreased along the longitudinal axis of the meniscus from both horns to the middle part of the body, which may guide future diagnostic methods and rehabilitation protocols.

**Electronic supplementary material:**

The online version of this article (10.1186/s12891-019-2706-x) contains supplementary material, which is available to authorized users.

## Introduction

The meniscus plays an important role in biomechanics, such as load bearing, load transmission and joint congruity. When the meniscus is torn, pain is usually the most common symptom [1]; however, the mechanism of pain remains unknown. After injury, some patients suffer acute pain immediately, while others do not. Meniscus injury in those patients without obvious symptoms cannot be detected until the injury progresses and a locked knee develops. Usually, a physical examination or magnetic resonance imaging is used to diagnose meniscus injury [2]. Degenerated diseases such as extrusion of the meniscal body can be asymptomatic and lead to osteoarthritis [3]. Therefore, it is necessary to determine the mechanism of pain. Understanding the distribution of nociceptors responding to noxious stimulus may provide answers to this question. Substance-P, a type of neuropeptide produced by nociceptors, can be detected by immunohistochemical staining and could possibly display the distribution of nociceptors [4].

Several studies have described the distribution of nociceptors in the meniscus. Assimakopoulos et al. demonstrated the presence of nociceptive innervation in the human meniscus [5]. Grey and Mine et al. described a rich supply of nerves and nociceptors on the anterior and posterior horns of menisci in a qualitative way [6, 7]. Ashraf et al. found nerve growth in human menisci, providing a potential mechanism for the genesis of pain in knee osteoarthritis [8]. However, heterogeneity in nociceptor quantity within different parts of the meniscus along the longitudinal axis remains inconclusive because no statistical comparisons have been performed. Furthermore, previous studies focus more on pathological or animal menisci, while intact human menisci from younger donors are a better choice to reveal the natural anatomy.

In general, pain can be divided into inflammatory pain and neuropathic pain [9]. In a torn meniscus, pain may originate from peripheral tissue inflammation such as secondary synovitis or from meniscus itself [10]. The authors hypothesized that meniscus lesions extending to the location of innervations and nociceptors, which are receptors in the meniscus that are specific to pain, might directly cause neuropathic pain, while meniscus tear occurring in the area where nociceptors are absent may later stimulate nociceptors in the synovia and joint capsule, resulting in delayed inflammatory pain.

Therefore, the purpose of this study was to evaluate the distribution characteristics of innervation and nociceptors in intact human menisci along the longitudinal axis. Apart from observing stained menisci under a microscope with H&E staining and improved chloride gold staining, the distributions of nociceptors among different parts of menisci were compared using a semi-quantitative immunohistochemical assessment. Finally, the relationship and mechanism between the location of pain and the location of the pathology, which could possibly guide clinical practice, was explored.

## Materials and method

All menisci were obtained from donors who underwent an amputation (1/11) due to accident or malignant tumour (10/11), with an intact knee joint and without any intraarticular symptoms. The time between ischaemia and dissection did not exceed 1 h. The age of the donors ranged from 22 to 39 years (9 males and 2 females, 28.45 ± 5.35 years). A total of 11 cases were included, containing 11 medial and 11 lateral qualified meniscus specimens. Ethics approval was obtained from the ethics committee of Sichuan University. The patients were informed about the research study and consented to participate. Verbal consent of all patients before surgery was obtained.

Two paired menisci (Group 1) chosen randomly from 11 pairs of menisci were stained by a modified gold chloride method. The remaining menisci (Group 2) were sectioned and stained by haematoxylin and eosin (H&E) and immunohistochemistry using a monoclonal antibody to substance-P.

In Group 1, the menisci were cut into small segments with a width of 5 mm and stained in bulk by the modified gold chloride method according to O’Connor and Gonzales [11]. The segments were frozen and consecutively sectioned on a sliding microtome (Leica, Wetzlar, Germany). Then, the sections were floated in anhydrous glycerol, mounted on slides, dehydrated and cover slipped. Finally, a light microscope (Olympus, Tokyo, Japan) was used to examine the serial sections to identify the morphology and distribution of the nerve endings.

All menisci specimens in Group 2 were fixed in 4% paraformaldehyde (0.1 mol/L) phosphate buffer (pH 7.3) for 24 h. Then, the specimens were cut into the anterior horn, posterior horn, and meniscal body with sections perpendicular to the longitudinal axis. The body part was equally divided into the anterior 1/3 of the body, the middle 1/3 of the body, and the posterior 1/3 of the body. A series of standard procedures were conducted to process these 5 portions of tissue segments, including sucrose dehydration, incubation with a mixed solution containing lemon juice, followed by formic acid and paraformaldehyde treatment for optimal tissue transparency, and paraffin for waxing and embedding (Leica, Wetzlar, Germany). Finally, the paraffin-embedded tissue samples were consecutively sectioned at 5 mm on a paraffin slicing instrument (Leica, Wetzlar, Germany) (Additional file 1).

The specimens were deparaffinized with 80% xylene and ethyl alcohol, rinsed with phosphate buffer solution (PBS, pH 7.4, ZSGB-Bio, Beijing, China), and stained with Mayer’s haematoxylin solution and 1% eosin alcohol solution according to standard H&E staining procedures. The serial sections were examined for histology with a light microscope. A streptavidin-biotin complex technique was employed for immunohistochemical staining. Sections were washed with PBS, soaked in 3% hydrogen peroxide to remove endogenous peroxidase activity (from blood cells), and then incubated with a monoclonal antibody to substance-P (Bioss Ltd., Beijing, China). The sections were then incubated with peroxidase-labelled streptavidin–biotin. 3,3′-Diaminobenzidine tetrahydrochloride dehydrate (DAB, ZSGB-Bio, Beijing, China) was used to visualize the localization of the antigens according to the manufacturer’s instructions. After washing in distilled water, the sections were counter-stained with haematoxylin and cover slipped. The nerve fibres positive for SP were observed using a light microscope and analysed semi-quantitatively with an imaging and processing system (Olymbus, Tokyo, Japan).

### Immunohistochemical evaluation

Ten visual fields from randomly selected slices were observed with a light microscope, and each field was photographed with a digital camera. On each photomicrograph, 2 senior pathologists blinded to the sample type identified and counted the number of fields exhibiting a positive reaction. Nerve fibres showing substance-P expression in sections from menisci were examined semi-quantitatively. The measurements were made in ten consecutive visual fields at 400 times magnification. In each field, the number of SP-positive fibres was determined using a three-grade scale according to Dariusz [12]: 0 – no positive nerves with substance-P in 10 fields; 1 – only one positive nerve fibre in the 10 visual fields; 2 – two or more fibres positive for substance-P in the 10 visual fields. Finally, the arithmetic mean of SP-positive fibres was calculated for the ten visual fields in each sample.

### Statistical methods

Single-factor analysis of variance was applied to analyse the differences among multiple groups of data. The normal distribution and homogeneity of variance were tested, and *p* values < 0.05 or 0.001 were considered significant. All data were analysed with the SPSS program (SPSS Statistics Version 25, Chicago, IL, USA).

## Results

### H&E staining

**Fig. 1:**
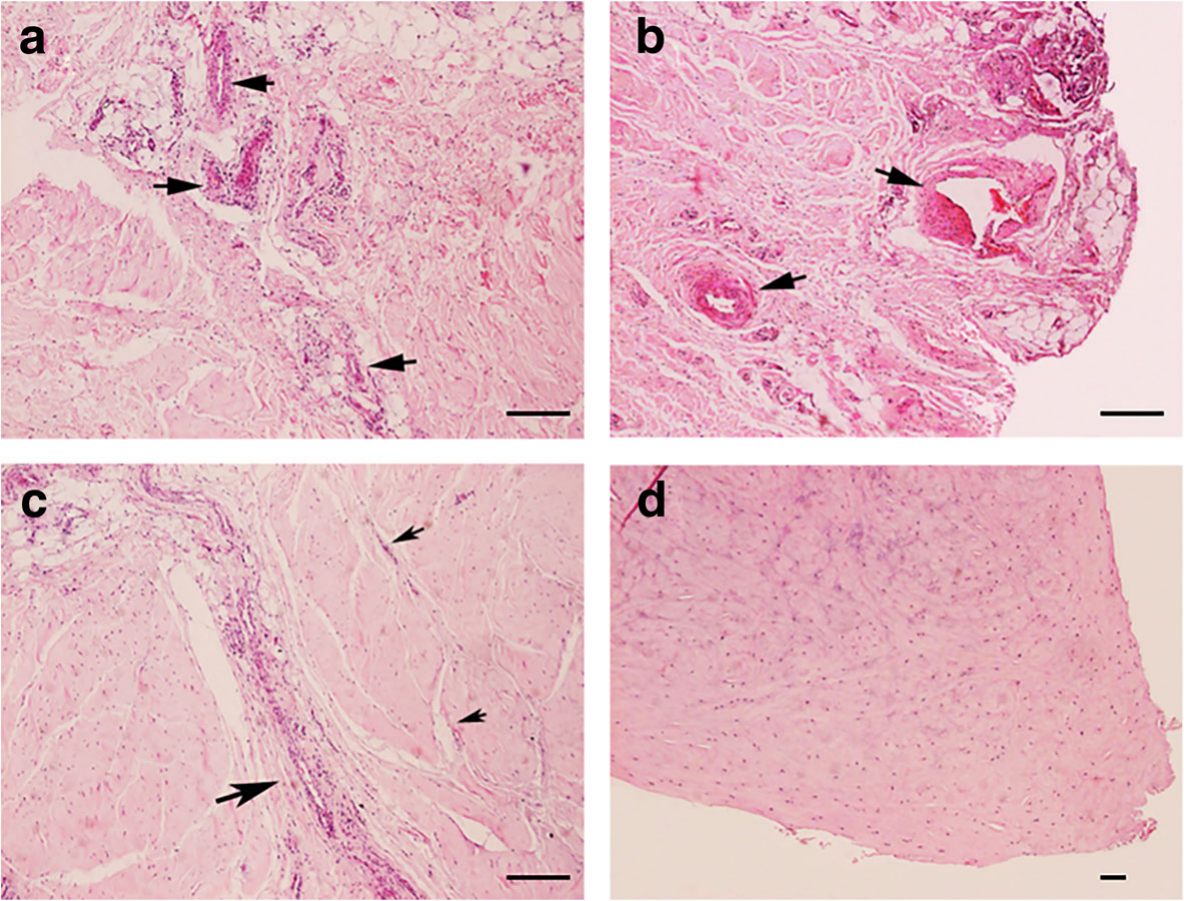
Meniscus H&E Staining under microscopy. **a, b**) Anterior horn (a) and posterior horn (**b**) show a large amount of loose connective tissue and nerve fibers accompanied by blood vessels (black arrow). **c**) Outer third body of meniscus shows collagen bundles separated by loose connective tissue (black arrows). **d**) Inner third body of meniscus shows no loose connective tissue

Under microscopy, the menisci displayed well-organized collagen bundles with meniscal cells. Chondrocytes were gathered into a round, oval or fusiform area and lined up between the bundles. Each portion of the meniscus showed a different histological structure (Fig. 1). Sections of the anterior horn and posterior horn showed a large number of nerve fibres accompanied by blood vessels. Specimens of the body sections showed an absence of loose connective tissue or nerve fibres in 70–90% of the width of the inner part of the menisci. In the remaining 10–30% width of the outer periphery, H&E staining demonstrated collagen bundles separated by loose connective tissue. A small number of nerve fibres were inserted between the bundles.

### Staining by a modified gold chloride procedure

Under the microscope, the background tissues appeared a light, pinkish/lavender colour. Nerve fascicles revealed intense black colour, which were surrounded by the epineurium, displaying a halo of light purple. The morphology and distributions of nervous tissue in the lateral and medial menisci were as follows.

**Fig. 2:**
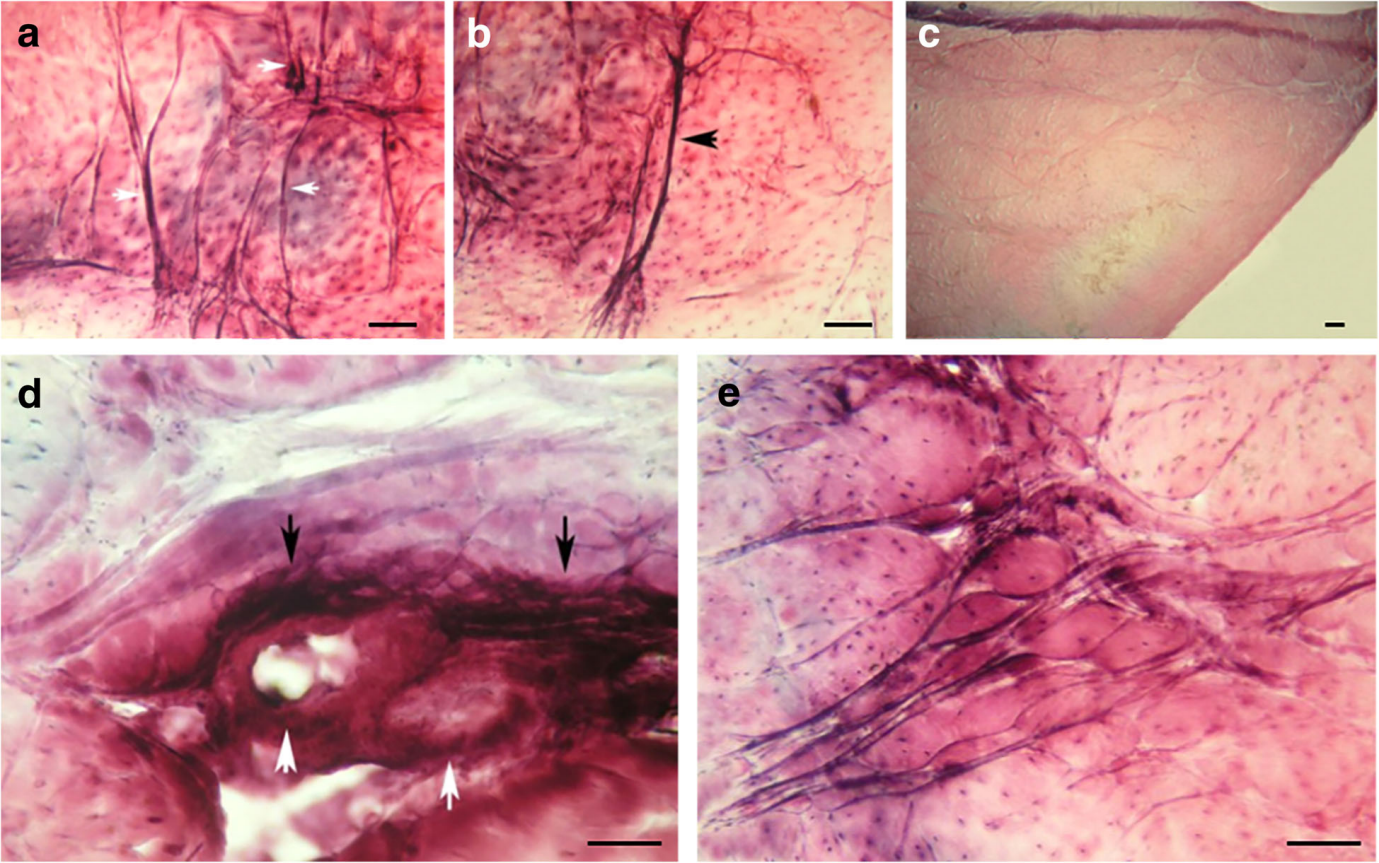
Gold chloride staining. **a**) The outer third of the menisci demonstrated abundant nerve fibers per low power field. And they were pilotaxitic, furcal and curly. **b**) Nerve fibers in the middle third were decreased in the number. And they became dendritic and furcal. **c**) At the central third, nerve tissues were absent. **d**) In the anterior horn, there were more abundant nerves fibers (black arrow) surrounding corresponding minutes blood vessels (white arrow). **e**) In the posterior horn,there were abundant nerves fibers as well

The outer third of the menisci demonstrated one or two free nerve endings per low-power field. Those in the middle third were fewer in the number, and in the inner third, free nerve endings were absent (Fig. 2a-c). There were more abundant nerves in the anterior horn and posterior horn than in the body. There was less nerve tissue in the body than in horns (Fig. 2d). In addition, most nerves, especially the major nerve, surrounded or ran parallel to blood vessels (Fig. 2d).

**Fig. 3:**
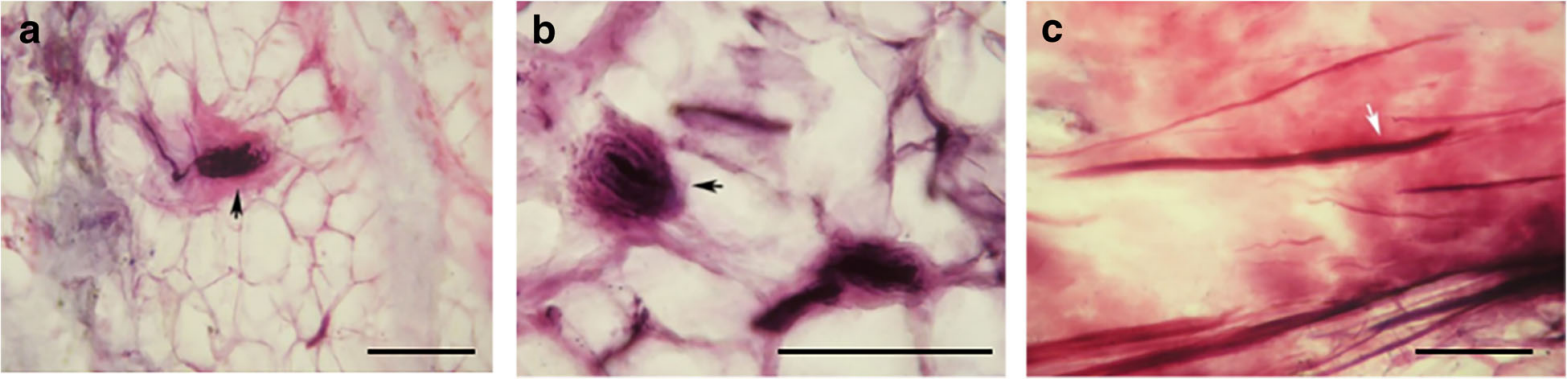
Gold chloride staining of mechanoreceptors and nociceptors. **a)** A Pacinian corpuscle was found in the anterior meniscal horn. **b**)A Golgi tendon organ was found in the posterior horn; **c**)Free nerve endings were present in the middle third of menisci

In the horns of the menisci, nerve fascicles entered the horns via the loose connective tissue septa that separated the collagen bundles. These fascicles consisted of nerve fibres ranging in size from small, unmyelinated fibres to large, myelinated fibres (Fig. 2d-e). In the body of the menisci, pilotaxitic, furcal and curly nerve fibres penetrated the outer third and extended to the junction of the middle and inner thirds where they became dendritic and furcal (Fig. 2a-c). In addition, corpuscles morphologically similar to the Ruffini corpuscle, Pacinian corpuscle and Golgi tendon organ, as identified by Freeman and Wyke [13], were found prevalently in the anterior and posterior meniscal horns and sparsely in the outer third (Fig. 3a-b). Furthermore, some free nerve endings were present in the outer third and middle third of the menisci, which were generally regarded as nociceptors (Fig. 3c). No difference in nerve distribution was observed between the medial and lateral menisci in the sections stained with gold chloride.

### Histological evaluation

**Fig. 4:**
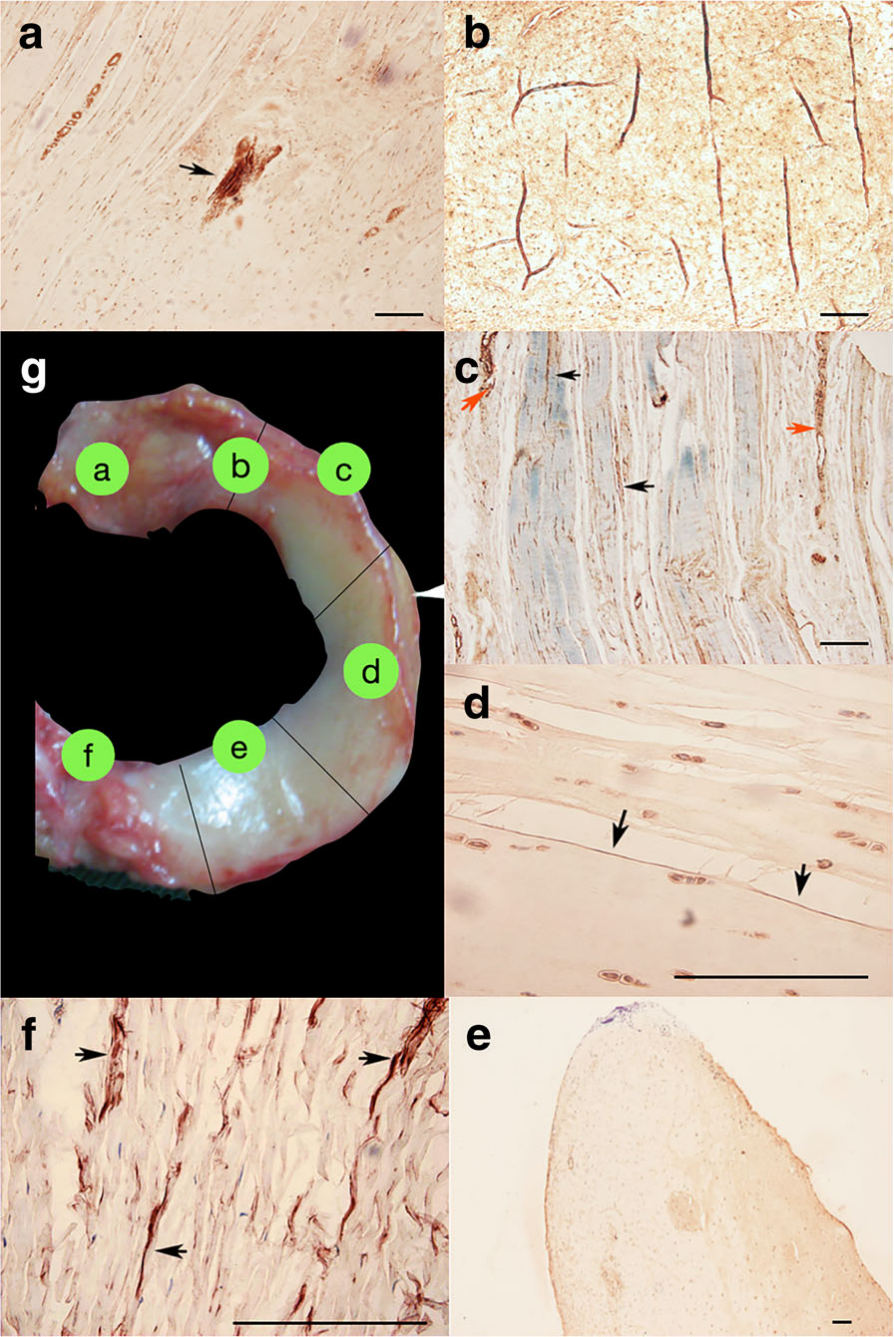
Different parts of meniscus immunohistochemically stained by substance-P. **a)** In anterior horn, SP+ fibers could be identified accompanied with blood vessels (black arrow); **b**) In inner third at the junction between anterior horn and anterior body, a small amount of isolated myelinated nerve fibers with SP+ can be seen; **c**). In outer third at the anterior body, SP+ fibers (black arrow) and blood vessels and connective tissue (red arrow) were observed; **d**). Thin, unmyelinated SP+ nerve fibers could be seen in outer third at the middle body; **e**). No nerve fibers or receptors were detected in the inner third of the posterior body of menisci; **f**). In inner third at the posterior horn, large amounts of SP+ fibers could be found; **g**). A medial meniscus was sectioned by five parts (black line) and specimens immunohistochemical stained from specific part were shown as (**a**)-(**f**)

Nerve fibres that were positive for substance-P (SP+), which is specific to nociceptive fibres, were detected in all five portions of the menisci, but the distribution density varied in each part (Fig. 4). In line with the results of gold chloride staining, nerve fibres and nociceptors that were SP+ were found mainly in the vascular outer third of the menisci; the positive area was wider in the anterior and posterior horns. Unmyelinated nerve fibres, with diameters of 0.5~1.5 μm, were more than myelinated fibres. In the intermediate middle body of the menisci, SP+ nerve fibres were not associated with blood vessels but ran through the loose connective tissue. No nerve fibres or receptors were detected in the inner third of the body of the menisci. However, in the inner third or avascular part at the junction between the anterior horn and the anterior body, a small number of isolated myelinated SP+ nerve fibres were observed.

SP+ fibres were most commonly associated with blood vessels. They inserted from where the meniscus attaches to the articular capsule, distributed in the outer periphery of the meniscus and sent some branches running radically towards the centre of the meniscus. Free nerve endings without branches were observed in connective tissue.

Compared with gold chloride staining, immunohistochemical staining showed that SP+ fibres were fewer in number, and the SP+ zone was narrower than the gold chloride-stained positive area. The patterns of innervation were more diverse in gold chloride staining than in SP staining. No Pacinian corpuscles, Ruffini corpuscles or Golgi tendon organs were identified on SP staining. Ranvier nodes on myelinated nerve fibres with a diameter of less than 5 μm of the biggest fibre were also much smaller than those identified with gold chloride staining.

### Semi-quantitative evaluation

**Fig. 5:**
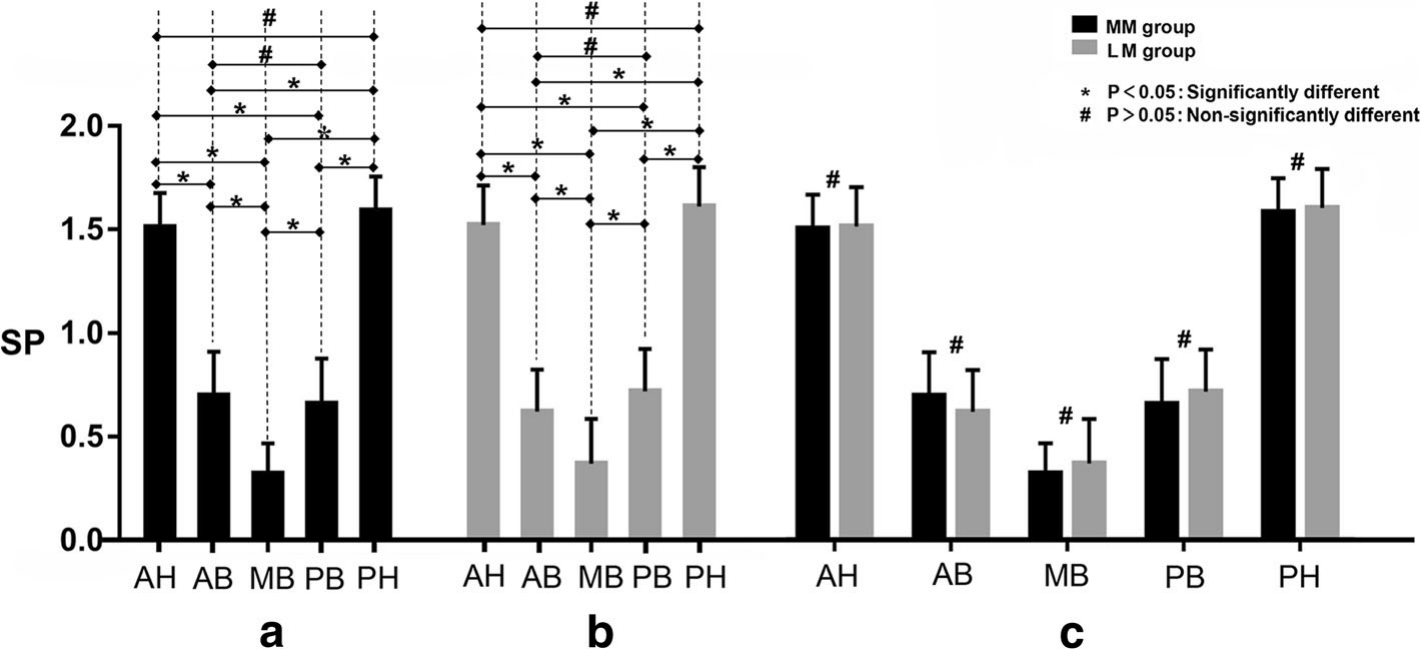
Summary of semi-quantitative assessment of density of substance-P positive nerve fibers. In both medial (**a**) and lateral (**b**) meniscus, SP+ fibers were greater in the anterior horn or posterior horn than in the all body parts; it was also greater in anterior body or posterior body than in the middle part. No significant difference was found between anterior horn vs posterior horn, anterior body vs posterior body and paired medial versus lateral meniscus (**c**). AH, Anterior Horn; AB, Anterior 1/3 Body; MB, Medial 1/3 Body; PB, Posterior 1/3 Body; PH, Posterior Horn; MM, Medial Meniscus; LM, Lateral Meniscus

The mean grades of ten consecutive visual areas at 400x magnification of all parts of each meniscus are shown in Table 1. In both the medial and lateral menisci, the number of SP+ fibres was significantly different among different parts of the meniscus (one-way ANOVA, *p* < 0.05). Comparison of the specimens in the distribution of substance-P nerve fibres from the same location between the paired medial and lateral menisci revealed no significant differences in any parts of menisci (*p* > 0.05), as shown in Fig. 5.

**Table 1:** Summary of the mean grade of substance-P positive fibres in ten visual fields

No.	Medial					lateral				
	Anterior horn	Anterior body	Middle body	posterior body	posterior horn	Anterior horn	Anterior body	Middle body	posterior body	posterior horn
1	1.50	0.70	0.50	0.40	1.90	1.60	0.50	0.20	0.70	1.60
2	1.70	0.60	0.40	0.70	1.50	1.80	0.80	0.80	0.40	1.20
3	1.60	0.90	0.20	0.90	1.50	1.50	0.40	0.20	0.90	1.80
4	1.50	0.30	0.10	1.10	1.80	1.50	0..60	0.20	0.60	1.60
5	1.20	0.50	0.30	0.60	1.60	1.30	0.60	0.50	0.80	1.60
6	1.40	0.60	0.40	0.60	1.40	1.80	0.90	0.40	0.60	1.30
7	1.70	0.90	0.50	0.80	1.50	1.40	0.90	0.10	1.00	1.40
8	1.60	1.00	0.40	0.50	1.70	1.60	0.70	0.40	1.00	1.70
9	1.60	0.70	0.10	0.50	1.60	1.40	0.50	0.60	0.50	1.30
10	1.30	0.80	0.30	0.50	1.40	1.70	0.30	0.30	0.70	1.60
Mean ± standard deviation	1.51 ± 0.17	0.70 ± 0.21	0.32 ± 0.15	0.66 ± 0.22	1.59 ± 0.17	1.56 ± 0.17	0.62 ± 0.22	0.37 ± 0.22	0.72 ± 0.20	1.51 ± 0.20

When making comparisons between each set of two parts among five areas (Fig. 5), no significant difference was found between the number of substance-P nerve fibres in the anterior horn versus the posterior horn or between the anterior body versus the posterior body of all menisci (*p* > 0.05). The mean number of substance-P fibres was greater in the anterior horn and posterior horn than in the anterior body, middle body, or posterior body (*p* < 0.05). Regarding the body of the meniscus, the mean number of fibres with substance-P was greater in the anterior body or posterior body than in the middle part (*p* < 0.05).

## Discussion

The present study assessed the meniscus with a more precise semi-quantitative method that focused on the exact distribution of nociceptors in specific areas of the meniscus and found that the density of nociceptors is statistically higher in both horns than in the body of meniscus.

The innervation of menisci has been investigated in several studies [5–7, 14–16]. Nerve fibres from the recurrent peroneal branch of the common peroneal nerve penetrate the peri-meniscal tissue in the outer third of the meniscus, with a higher density in both horns. In the body of the meniscus, the number of neural elements decreases from the outer periphery towards the centre, similar to the vascular anatomy pattern. Nociceptors, with a similar distribution pattern of nerve fibres, were found within the meniscus. The results of the present work were consistent with the qualitative observations. In addition, this study clearly shows that the number of nociceptors decreases statistically along the longitudinal axis of the meniscus from both horns to the middle part of the body.

Zimny et al. reported [17] that neural elements are in greater concentration in the posterior horn than in the anterior horn. In the present work, however, no significant difference was found between the anterior and posterior horns, between the anterior and posterior bodies or between the medial and lateral menisci, based on the results of both modified gold chloride and substance-P immunohistochemical staining. The discrepancy might be explained by the methodological differences. Quantitative assessment in this study specifically to SP+ nociceptors described this issue more precisely than previous studies did.

Pain is the most common symptom when patients have an injured meniscus and subsequently seek medical service. Despite the availability of radiological techniques, medical history and physical examinations are still essential for clinical diagnosis. However, pain derived from the meniscus could be mimicked by some other knee pathology. Iliotibial band syndrome, proximal tibiofibular joint instability, snapping biceps femoris or popliteus tendons, and peroneal nerve compression syndrome or neuritis present with similar clinical findings as those associated with a lateral meniscus tear and can mimic a meniscus injury [18].

In a pilot study [19], Campbell et al. found no direct correlation between the location of pain and the location of an injured meniscus. The present results suggest that, based on the patient’s history of trauma, pain at the time of injury was derived from the innervated area of the meniscus, including the anterior horn, posterior horn and outer third of the body. The location of acute pain of this type is closely related to the underlying pathology and could consequently guide the diagnosis. However, delayed pain might indicate that the lesion occurred in an area where nociceptors are rare or absent (i.e., the inner part of the meniscal body). Sensory pain was not generated immediately after injury until secondary synovitis developed [7]. In this case, patients with meniscus injury without pain symptoms may be undiagnosed.

The anterior horn of the lateral meniscus [20] and the posterior horn of the medial meniscus are the most frequently injured areas with symptoms due to their anatomical and biomechanical features, which is also consistent with the clinical observations. This study, though not reaching a significant difference, shows a trend towards a higher density of nociceptors in the anterior horn of the lateral meniscus and the posterior horn of medial meniscus than in the posterior horn of the lateral meniscus or the anterior horn of the medial meniscus, respectively. Patients with a torn meniscus in these parts usually complain of sharp pain due to stress on the tibiofemoral joint. Interestingly, another common injured structure of meniscus [21], the meniscal root, may present no pain upon clinical observation when completely torn. One possible explanation is the lack or absence of mechanical stimuli if completely torn, as nociceptors detected in this study are a type of unmyelinated C-fibre [9], which respond to noxious stimuli of a mechanical (tactile and pressure), thermal or chemical nature [22]. In addition, partial meniscectomy is thought to be a risk factor for osteoarthritis due to the uneven distribution of the knee joint load [23]. With the knowledge of the distribution of the nerve fibres on the meniscus, individual neuromuscular training could be developed according to the removed portion of the meniscus, leading to a more precise rehabilitation programme [24].

Patients with grade 2 meniscal injury may suffer from pain, but the indications for surgery remain controversial [25]. According to H&E staining and gold chloride staining, most nerve fibres and nociceptors run along with blood vessels. From clinical experience, patients with grade 2 meniscal injury may present tenderness in the tibiofemoral joint line with or without subjective pain. As tissue in the vascular zone has a higher potential to heal [26], it seems that symptomatic grade 2 meniscus injury could and should be treated if any promising operative or semi-invasive methods are proposed in the future.

The present study used immunohistochemical staining instead of directly counting the number of nociceptors in serial slices of a single meniscus, which may contribute to fewer false-positive and false-negative results because the nociceptors may resemble blood vessels in some slices. Furthermore, the mean age of the patients from whom samples were acquired is relatively younger than the age of donors in previous studies, which is more practical because traumatic tears usually occur in younger, active individuals [27]. A potential weak point was that the sample size was relatively small, which possibly explains why a significant difference was not reached in the anterior versus posterior horns as described previously.

## Conclusion

The present study revealed that nociceptive nerve fibres are distributed mostly in both horns and the outer third body of an intact meniscus, which provides a heightened awareness when dealing with a patient complaining of knee pain with suspected meniscus tears.

## Additional file

Additional file 1: Flowchart of experimental procedures. (PNG 123 kb)

## Data Availability

The datasets used and analysed during the current study are available from the corresponding author on reasonable request via yipenglin.md@gmail.com or liqimm@yahoo.com.

## Abbreviations

H&E: Hematoxylin-Eosin
SP: Substance-P
SP +: Substance-P positive
