# Supplementary figures and images for: Innervation of nociceptors in intact human menisci along the longitudinal axis: semi-quantitative histological evaluation and clinical implications
Source: BMC Musculoskelet Disord. 2019 Jul 22;20:338. doi: 10.1186/s12891-019-2706-x (PMC6647164; doi:10.1186/s12891-019-2706-x)

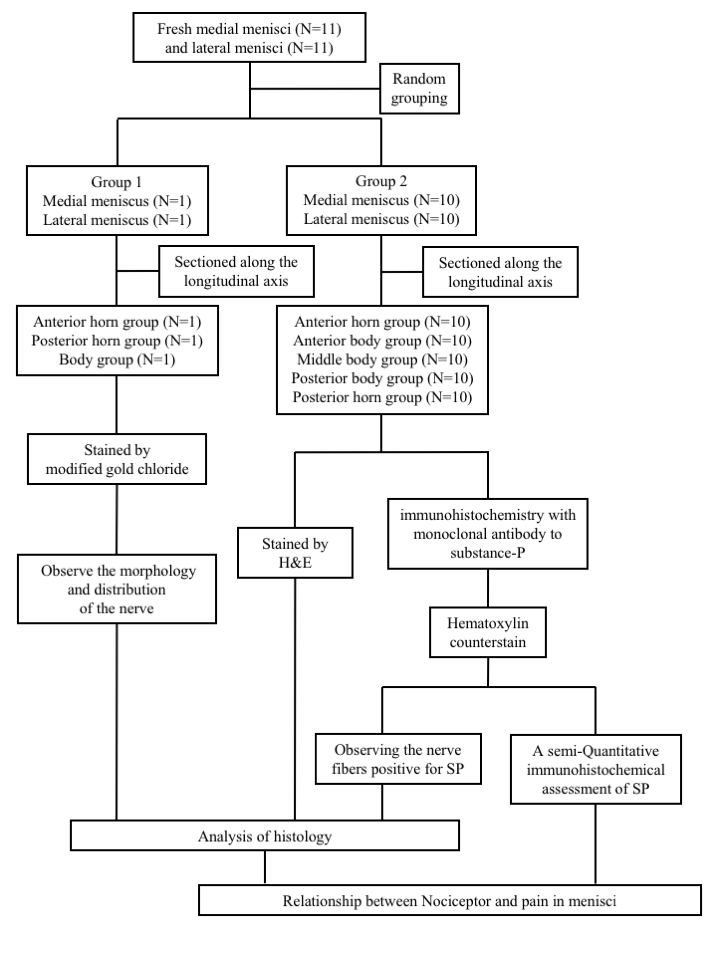

Supplement: Supplementary file 1 — Flowchart of experimental procedures. (PNG 123 kb) [file 12891_2019_2706_MOESM1_ESM.png]
